# Efficiency of combined systemic glucocorticoids and narrowband ultraviolet B phototherapy in lymphocytic-variant hypereosinophilic syndrome

**DOI:** 10.1016/j.jdcr.2026.05.003

**Published:** 2026-05-11

**Authors:** Yasutoshi Hida, Tomoko Hara

**Affiliations:** a Division of Dermatology, Tokushima Red Cross Hospital, Komatsushima, Tokushima, Japan; b Division of Hematology, Tokushima Red Cross Hospital, Komatsushima, Tokushima, Japan

**Keywords:** lymphocyte immunophenotyping, lymphocytic-variant hypereosinophilic syndrome, narrowband ultraviolet B phototherapy, systemic glucocorticoid therapy, T-cell receptor β gene rearrangements, thymus and activation-regulated chemokine

## Introduction

Hypereosinophilic syndromes (HES) are rare disorders characterized by persistent peripheral eosinophilia (absolute eosinophil count [AEC]≥1500/μL) with organ involvement due to eosinophil infiltration. HES are heterogeneous and divided into 3 groups according to mechanisms underlying eosinophil expansion: (1) neoplastic, with clonal proliferation of eosinophils, such as chronic eosinophilic leukemia commonly with FIP1-like 1/platelet-derived growth factor receptor alpha (*FIP1L1–PDGFRα*) fusion gene; (2) reactive, in which increased production of eosinophilopoietic factors leads to polyclonal eosinophil expansion; and (3) idiopathic, in which the mechanism driving eosinophilia remains unclear.^1^ Patients with clonal T-cells, which are the source of eosinophilopoietic cytokines, are classified as having reactive HES.^1^ This subtype, called lymphocytic-variant HES (L-HES), is characterized by recurrent skin manifestations and occasional severe and persistent pruritus, which is often resistant to systemic glucocorticoid (GC) therapy.^2^

Herein, we report 3 cases of L-HES treated with combined systemic prednisone and narrowband ultraviolet (UV) B (NB-UVB) phototherapy and reveal the potential positive effects of NB-UVB phototherapy on intractable pruritus.

## Case series

### Case 1

**Fig 1:**
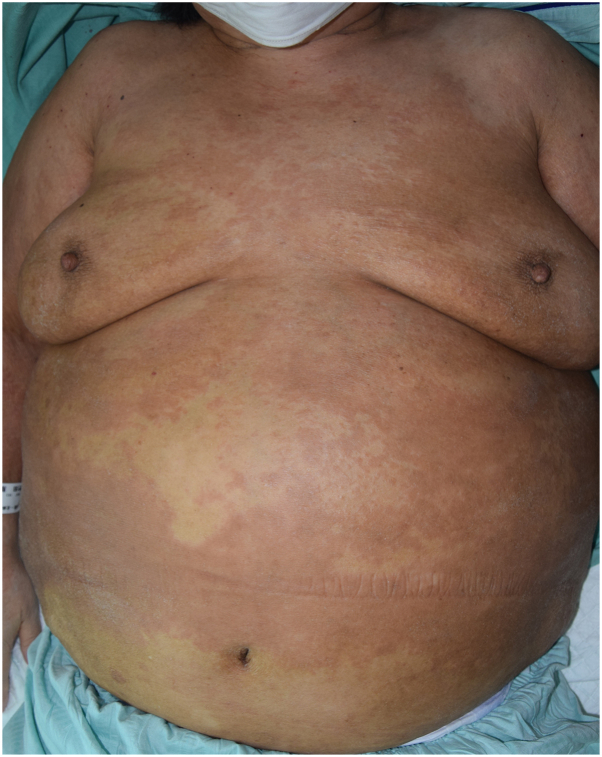
Extensive erythematous patches and plaques are observed throughout the body.

**Fig 2:**
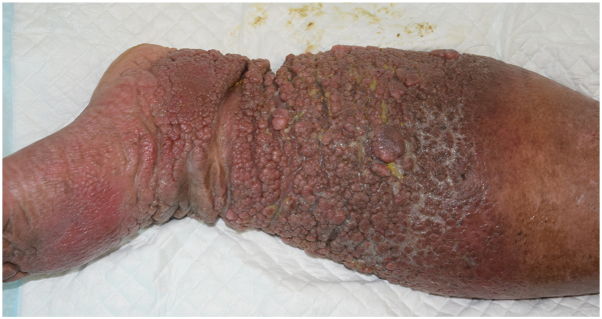
Papillomatous proliferation and edema are noted on the lower left leg.

A 64-year-old woman presented to the emergency department of our hospital with a chief complaint of dyspnea, generalized pruritic skin lesions, and general fatigue persisting for 1 year. She did not seek hospital care since symptom onset and had not taken any medications. Skin examination revealed extensive erythematous patches and plaques throughout the body (Fig 1). She had papillomatous and edematous skin proliferation on the lower left leg (Fig 2), due to deep vein thrombosis that was identified by ultrasonography of lower extremity veins. Skin biopsy of the erythematous patch showed slight hydropic degeneration of the basal cells and perivascular lymphocytic infiltrations mixed with eosinophils in the upper dermis. Blood tests revealed marked eosinophilia (26.2%; AEC, 6737/μL) and impaired renal function (serum creatinine, 3.09 mg/dL; blood urea nitrogen, 66 mg/dL). Other laboratory tests demonstrated a soluble interleukin (IL)-2 receptor level of 1885 (normal, 231-646) U/mL, total immunoglobulin E (IgE) level of 5496 (normal, <170) IU/mL, and thymus and activation-regulated chemokine (TARC) level of 17,246 (normal, <450) pg/mL. Tests for autoimmune markers, including antineutrophil cytoplasmic antibody, stool tests for ova and parasites, and testing for human immunodeficiency virus (HIV) were negative. Bone marrow aspiration revealed eosinophilic hyperplasia without abnormal cells. Fluorescence in situ hybridization result for the *FIP1L1–PDGFRα* fusion gene was negative. Gene mutation analyses of Janus kinase 2, calreticulin, and nucleophosmin1 were negative, and the Philadelphia chromosome was not detected. Polymerase chain reaction (PCR) analysis of T-cell receptor (TCR)-β gene rearrangements identified a monoclonal population, and flow cytometry showed an aberrant circulating CD3−CD4+ T-cell population (lymphocytes, 1.7%). Overproduction of IL-5 was not observed. No abnormalities were detected on thoracoabdominal computed tomography, excluding axillary and inguinal lymphadenopathies.

The patient was diagnosed with L-HES and started on oral prednisolone (PSL) 0.5 mg/kg daily, warfarin, epinastine hydrochloride 20 mg/d, and topical steroid (betamethasone butyrate propionate). Although these treatments improved skin lesions and laboratory values, pruritus persisted. NB-UVB phototherapy was performed once daily, 3 times a week at an initial dose of 0.4 J/m^2^ that increased to 0.5 J/m^2^. After 10 treatment sessions, pruritus and skin lesions completely resolved. Although the PSL dose was tapered and then discontinued, pruritus eruption did not relapse, and the AEC and TARC values remained normal. After the disappearance of papillary hyperplasia in the left lower leg, warfarin was discontinued. NB-UVB phototherapy was continued once every 2 weeks for maintenance.

### Case 2

**Fig 3:**
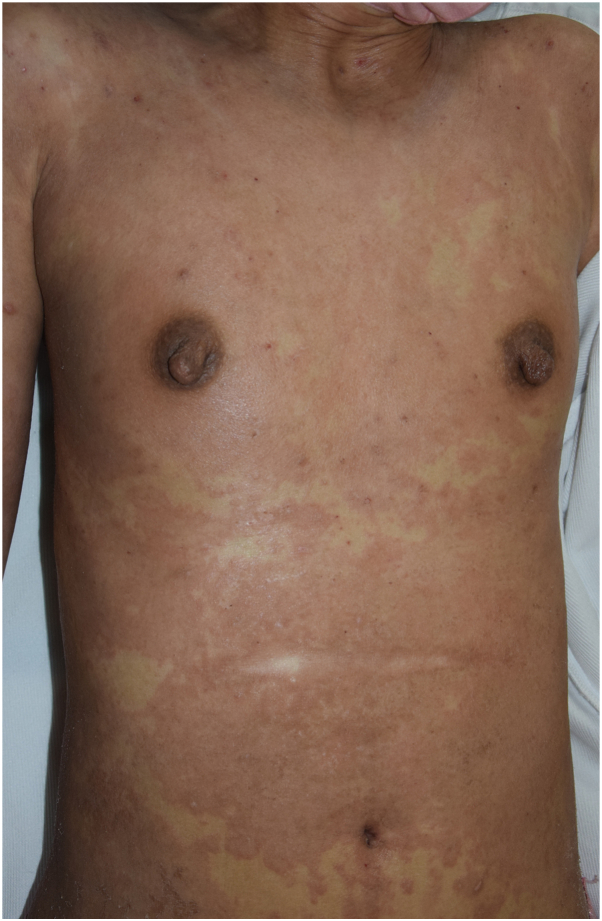
Exfoliative erythematous patches are scattered throughout the body.

A 46-year-old woman had a 1-year history of pruritic and erythematous eruptions on the trunk. She was diagnosed by a local physician with drug-induced eruption, which was attributed to analgesic use (ibuprofen, allylisopropylacetylurea, and anhydrous caffeine) for chronic headaches. The withdrawal of the analgesics, topical corticosteroids (betamethasone butyrate propionate), and bepotastine besilate (20 mg/d) did not resolve the symptoms. The patient was prescribed PSL (20 mg/d) for 10 days; however, after discontinuation, she developed a fever (38 °C), a worsening skin rash, and vomiting. The patient was admitted to our hospital. Skin examination revealed exfoliative erythematous patches scattered throughout the body, as well as cervical, axillary, and inguinal lymphadenopathies (Fig 3). Skin biopsy showed perivascular lymphocytic infiltration along with eosinophils in the upper dermis. Laboratory examination revealed marked eosinophilia (70.0%; AEC, 20,174/μL), lactate dehydrogenase level of 713 (normal, 124-222) U/L, and TARC level of 8065 pg/mL. The total IgE level was normal, and stool samples for ova and parasites were negative. Bone marrow aspiration revealed eosinophilic hyperplasia without abnormal cells. Fluorescence in situ hybridization result for the *FIP1L1–PDGFRα* fusion gene was negative. Biopsy of the right inguinal lymph node was performed, and the pathological examination revealed proliferative lesions of the lymphoid tissue with eosinophilic infiltration consistent with dermatopathic lymphadenopathy. Immunohistochemical staining of the lymph nodes did not reveal any lymphocytes with abnormal surface markers. PCR analysis of TCR-β gene rearrangements using resected the lymph node identified a monoclonal population. However, peripheral blood flow cytometry of circulating aberrant T-cells expressing CD3− CD4+ was not performed. Thus, in patients with HES, a monoclonal T-cell population might produce eosinophilopoietic cytokines.

Oral PSL (60 mg/day), bepotastine besylate (20 mg/day), and topical steroids (clobetasol propionate) were administered. These treatments normalized the AEC; however, intractable severe pruritus persisted. Thus, NB-UVB phototherapy was initiated once daily, 5 times a week, at a dose of 0.4 J/m^2^. After 9 treatment sessions, the pruritus subsided. The PSL dose was tapered and discontinued. After 30 NB-UVB treatment sessions, pruritus completely subsided, and all skin lesions resolved. Thus, NB-UVB phototherapy was discontinued.

### Case 3

**Fig 4:**
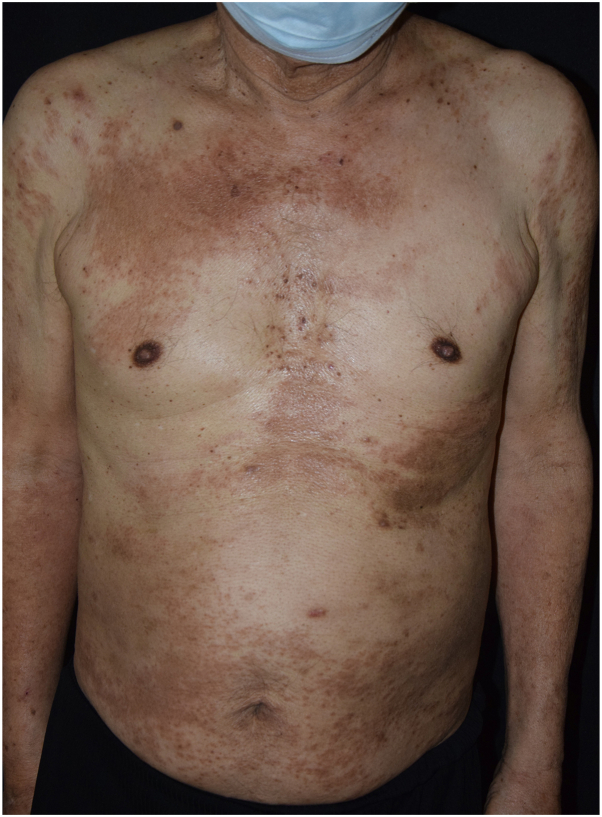
Disseminated reddish-brown papules and confluent plaques are seen throughout the body.

A 68-year-old man was referred to the hematology department of our hospital for eosinophilia (60.0%; AEC, 10,500/μL) and pruritic skin rash on the extremities. The patient’s medical history included diabetes mellitus, hypertension, and chronic kidney disease, with no history of drug hypersensitivity. The patient was diagnosed with idiopathic HES. Bone marrow aspiration revealed eosinophilic hyperplasia without abnormal cells, thoraco-abdominal computed tomography revealed pericardial effusion, and tests for chromosomal abnormalities, autoimmune markers, HIV, *FIP1L1–PDGFRα* fusion gene, and stool parasites were negative. He refused systemic steroid therapy because of concerns regarding side effects. After 4 years, his skin rash worsened, and pruritus became intractable, which led to his referral to our department. On skin examination, 1 to 5 mm reddish-brown papules and confluent plaques were widely seen on his body (Fig 4). Skin biopsy showed perivascular and interstitial infiltration of lymphocytes and eosinophils in the upper dermis. Laboratory analysis revealed an IgE level of 472 IU/mL and TARC level of 12,710 pg/mL. In tests using peripheral blood, PCR analysis of TCR-β gene rearrangements identified a monoclonal population, and flow cytometry showed aberrant circulating CD3− CD4+ T-cells (0.3% of lymphocytes). The patient was diagnosed with L-HES, and NB-UVB phototherapy was started once weekly at an initial dose of 0.4 J/m^2^, which increased to 0.5 J/m^2^. The itching subsided to a tolerable level, and the AEC decreased; however, the eruptions only slightly improved. He subsequently suffered a cerebral infarction, and oral PSL was started at 30 mg/day, in addition to NB-UVB phototherapy. This treatment led to the complete clearance of his skin lesions and resolution of the pruritus. The PSL dose was then tapered to 3 mg/day. NB-UVB phototherapy was continued once every 2 weeks for maintenance.

## Discussion

L-HES is characterized by the overproduction of eosinophilopoietic cytokines by an aberrant T-cell population demonstrated by immunophenotyping, mainly by CD3−CD4+ T-cells.^1^ Diagnostic assessment of L-HES is performed by lymphocyte immunophenotyping using peripheral blood and analysis of TCR gene rearrangement patterns by PCR.^3^ According to the World Health Organization, the finding of isolated T-cell clonality by PCR is insufficient to diagnose this variant.^3^ Carpentier et al reported that aberrant CD3−CD4+ T-cells accounted for ≤2% of total lymphocytes in approximately 50% of patients with L-HES, with the lowest observed percentage being only 0.15%.^1^ Most patients with L-HES showed significantly increased serum TARC levels, implying that abnormal T-cells enhanced the production of type 2 cytokines, leading to eosinophil expansion.^1^ Additionally, the serum TRAC level may be a useful diagnostic biomarker.^1^^,^^3^ In this report, PCR analysis of the TCR rearrangements identified a monoclonal T-cell population in all 3 patients. In cases 1 and 3, CD3−CD4+ aberrant T-cells were identified by lymphocyte immunophenotyping using peripheral blood. However, in case 2, peripheral blood flow cytometry to detect circulating aberrant T-cells expressing CD3− CD4+ was not performed before starting systemic GCs because of our limited understanding of the disease at that time. Based on the clinical symptoms, TCR rearrangement results, high TARC levels, and clinical course, all 3 cases were considered L-HES.

Systemic GC therapy is the first-line treatment for HES, and patients with L-HES respond well to GCs. Compared with idiopathic HES, L-HES may cause pruritic skin lesions that evolve accordingly and cause substantial morbidity. Additionally, owing to the resistance of pruritus to GCs and/or its side effects, adjunctive therapies are needed. Currently, several adjunctive therapies have been explored. Trials on interferon-alpha, monoclonal antibodies targeting IL-5, other immunosuppressive or cytotoxic compounds, and phototherapy have been conducted.^1^ Some studies have shown that phototherapy is an effective adjunctive therapy for HES.^4^, ^5^, ^6^, ^7^, ^8^ In English-language case reports, with detailed clinical course, phototherapy was attempted in 10 patients with HES.^4^, ^5^, ^6^, ^7^, ^8^, ^9^, ^10^, ^11^, ^12^, ^13^ The reported types of phototherapies include broadband UVB (BB-UVB), high-dose UVA1, psoralen with UVA (PUVA), and NB-UVB. Among the patients, only 1 achieved a satisfactory result with phototherapy alone using high-dose UVA1.^4^ In 5 patients, phototherapy alone was insufficient, which comprised PUVA in 3 patients, BB-UVB in 1, and NB-UVB in another one.^9^, ^10^, ^11^ Conversely, 4 patients received systemic GCs combined with phototherapy, and all achieved complete resolution, leading to the reduction of the systemic GC dose or its discontinuation.^5^, ^6^, ^7^, ^8^ Among them, reports of the 2 patients emphasized the effectiveness of phototherapy in treating severe pruritus.^5^^,^^8^ The HES type was unknown in 3 of the 4 cases because of older publication dates. However, a recent report on L-HES revealed that NB-UVB phototherapy was highly effective in relieving pruritus that was unresponsive to systemic GCs.^5^ Because NB-UVB phototherapy suppresses the production of Th2 chemokines, including TARC, its therapeutic effect on L-HES may result from the inhibition of these chemokines. Although NB-UVB phototherapy can alleviate pruritus and serves as useful method for tapering GC, it alone could not completely relieve pruritus, and its effect on skin eruptions was limited in case 3. Therefore, L-HES requires systematic GC therapy, and NB-UVB phototherapy may be useful as an adjunctive therapy for pruritus and tapering GC.

Recent reports have demonstrated the effectiveness of dupilumab in patients with HES.^9^^,^^14^ Dupilumab, a human monoclonal antibody, binds to the IL-4 receptor and inhibits the signaling of the Th2 chemokines IL-4 and IL-13. The effect of dupilumab on HES may be ascribed to the suppression of Th2 cytokines. However, the reported adverse effects were attributed to the increased use of dupilumab, including the development of cutaneous T-cell lymphoma (CTCL).^15^ Mara et al reported that 3 patients with CTCL who were treated off-label for severe pruritus experienced leukemic transformation and were subsequently diagnosed with Sézary syndrome.^15^ Two of them died from disease progression.^15^ Notably, L-HES carries an inherent risk of T-cell lymphoma, which eventually develops in approximately 10% of patients.^1^

### Declaration of generative AI and AI-assisted technologies in the writing process

AI was not used in the composition of this manuscript.

## Conflicts of interest

None disclosed.
